# Intestinal microbiota and antibiotic-associated acute gastrointestinal injury in sepsis mice

**DOI:** 10.18632/aging.202768

**Published:** 2021-03-26

**Authors:** Ci Han, Nana Guo, Yue Bu, Yahui Peng, Xueting Li, Xiaohui Ma, Mengyuan Yang, Xiaonan Jia, Jin Zhang, Xiaowei Liu, Kaijiang Yu, Changsong Wang

**Affiliations:** 1 Department of Critical Care Medicine, The First Affiliated Hospital of Harbin Medical University, Harbin Medical University, Harbin 150001, Heilongjiang, China; 2 Department of Critical Care Medicine, Harbin Medical University Cancer Hospital, Harbin Medical University, Harbin 150081, Heilongjiang, China

**Keywords:** intestinal microbiota, 16S rRNA, untargeted metabolomics, AGI, sepsis

## Abstract

Background: To investigate the changes of intestinal microbiota and metabolites in sepsis mice with acute gastrointestinal injury before and after the use of antibiotics, and to explore the possible effects of these changes on the body.

Methods: Twenty-four 6-8-w-old SPF-grade C57BL/6J male mice were selected, and the mice were randomly divided into three groups. The mice were treated by tail vein injection for 3 days. The intestinal motility of mice after administration was detected. The mice feces were collected for 16S rRNA and Untargeted metabonomics detection.

Results: The use of antibiotics in sepsis mice can change the composition of intestinal microbiota and metabolites. LD3, AD3 and LAD3 samples had significant differences in bacterial species. *Desulfovibrio* was the species with a significant difference in LAD3. In addition, we found that the composition of those intestinal microbiota were correlated with changes in intestinal motility. The untargeted metabolomics analysis showed that the fecal metabolites of LD3 and LAD3 samples were significantly different. In addition to the basic metabolites, Benzoic acid and 4-Hydroxybenzoic acid were also found, and *Desulfovibrio* was associated with them.

Conclusions: The use of antibiotics in sepsis mice can lead to changes in the intestinal microbiota and metabolite levels, which may be related to the severity of acute gastrointestinal injury in sepsis mice. Inhibiting *Desulfovibrio* in the intestine and using Benzoic acid and 4-Hydroxybenzoic acid as a marker for the production of *Desulfovibrio* may reduce the inflammatory degree of acute gastrointestinal injury in sepsis.

## INTRODUCTION

Sepsis is a serious infection that specifically induces an inflammatory response and endangers the life of patients [1]. Gastrointestinal dysfunction is a common problem in critically ill patients. Studies have shown that approximately 60% of patients show at least one gastrointestinal symptom during intensive care unit (ICU) admission [2]. Increasing evidence shows that gastrointestinal dysfunction is associated with high mortality in patients with sepsis [3, 4]. However, to date, although there are many hypotheses about the mechanism of sepsis and gastrointestinal dysfunction, such as sepsis having an initial impact on the gut so that the gut plays an initiating role in critical diseases, there is still no strong evidence for this statement [5, 6]. In 2012, the European Society of Intensive Care Medicine (ESICM) defined acute gastrointestinal injury (AGI) as gastrointestinal dysfunction caused by acute diseases in severe patients. It is important to monitor gastrointestinal status in critically ill patients, as patients with AGI have a high mortality rate [7, 8].

The use of antibiotics often causes gastrointestinal adverse events in patients, and studies have shown that this situation is usually attributed to changes in the composition and diversity of intestinal microorganisms, known as ecological disorders [9]. Although there are individual differences in the composition of the intestinal microbiota in healthy individuals, the specific functions of the symbiotic intestinal microbiota play an important role in maintaining homeostasis in the host [10]. The transition from a normal intestinal microbiota to a dysfunctional microbiota can lead to the loss of the beneficial metabolic functions of the intestinal microbiota, such as polysaccharide digestion, energy metabolism, and short-chain fatty acid (SCFA) synthesis, resulting in a lack of basic nutrients for maintaining intestinal function [11, 12]. Epidemiological investigation showed that intestinal microecology and bacterial translocation increased after antibiotics use [13, 14], which could increase susceptibility to intestinal inflammation [15, 16]. However, at present, antibiotics are used in the treatment of sepsis patients, so the risk of antibiotic-related gastrointestinal adverse events in patients with sepsis is increased. Therefore, we suspect that the use of a large number of antibiotics in patients with sepsis may cause intestinal microecological disorder, which is involved in the occurrence and development of AGI in patients with sepsis, affecting sepsis patient mortality.

To explore the correlation between the acute gastrointestinal injury in sepsis and intestinal microbiota and metabolites, we conducted relevant experiments. The results showed that the use of antibiotics in septic mice destroyed the intestinal microbiota and affected the composition of intestinal metabolites. This change may be associated with acute intestinal injury during sepsis. A bacterium associated with acute intestinal injury in sepsis and its related markers were found.

## RESULTS

To study the effect of antibiotics on the intestinal microbiota, we used imipenem/cilastatin, a common antibiotic formulation in the ICU, to intervene in mice treated with LPS.

**Figure 1:**
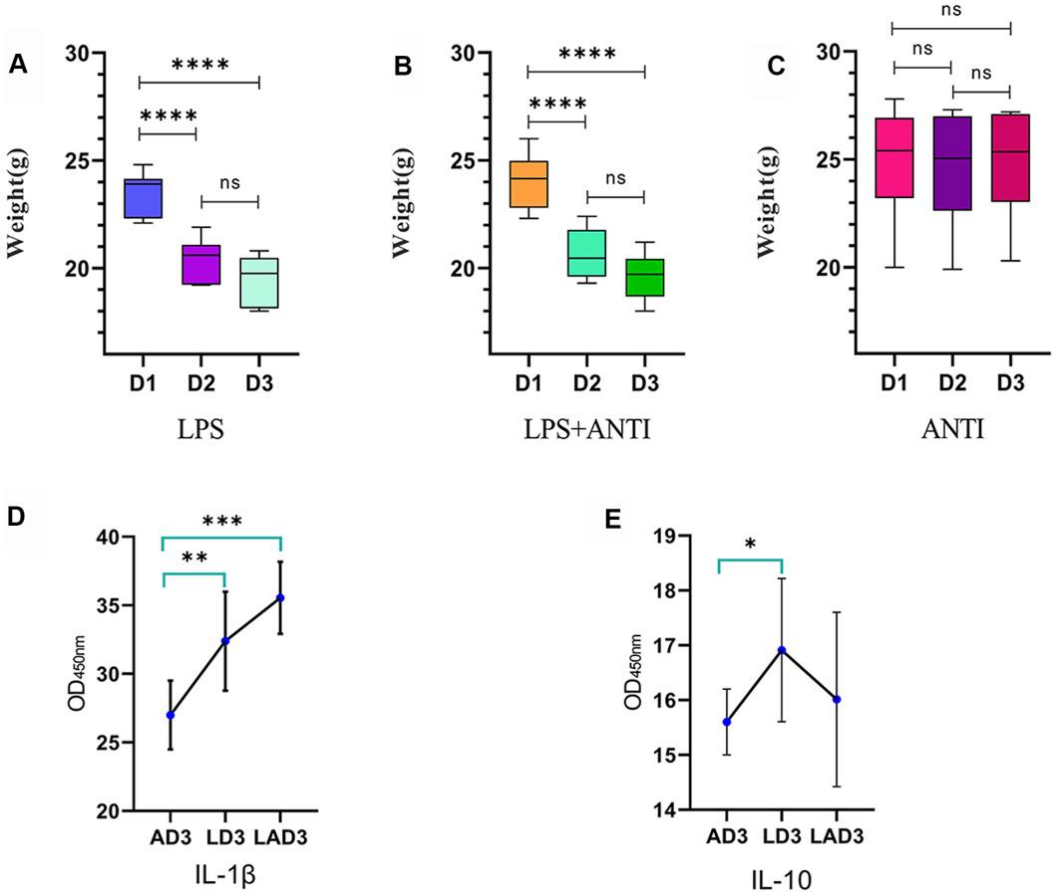
**Weight change trend of LPS group, LPS + antibiotic group and antibiotic group before and after administration.** (**A**) The weight change trend of mice in LPS group from day 1 to day 3 (D1, D2, D3), and the levels of IL-1 β and IL-10 in intestinal tract (AD3, LD3 and LAD3) on the third day after administration were detected by ELISA. (**B**) The body weight of mice in LPS + antibiotic group changed from day 1 to day 3 (D1, D2, D3). (**C**) The weight change trend of mice in antibiotic group from day 1 to day 3 (D1, D2, D3). (**D**) The levels of IL-1 β in AD3, LD3 and LAD3 groups were compared. *P<0.05, **P<0.01, ***P<0.001, ns>0.05. (**E**) The levels of IL-10 in AD3, LD3 and LAD3 groups were compared.*P<0.05, **P<0.01, ***P<0.001, ns>0.05.

(1) The three different interventions led to changes in mice weight (Figure 1 and Table 1) and defecation. We found that the mice who were administered LPS lost weight (Figure 1A), were mentally depressed, exhibited changes in the shape and character of feces, and displayed formation of pseudomembranes and blood in the stool; when the mice were treated with antibiotics alone, the body weight was not significantly changed (Figure 1C), the feces were golden and soft, and the feces were not formed. However, the weight loss of LPS-administered mice treated with antibiotics was the most obvious (Figure 1B), and the feces were dry, dark and even difficult to defecate. The levels of IL-1β and IL-10 in the 3rd day after continuous administration of antibiotic (AD3), the 3rd day after continuous administration of LPS (LD3) and the 3rd day after continuous administration of LPS and antibiotic (LAD3) groups were different (Figure 1E, 1F). We think that LPS caused damage to the intestinal tract, resulting in different inflammatory factors, and the use of antibiotics had different effects on the levels of inflammatory factors in sepsis mice. Compared with LD3, antibiotic use may increase IL-1β or decrease IL-10 in LAD3.

**Table 1:** The changes of body weight during the three groups of LPS, LPS and LPS+antibiotic were compared.

**Group**	**D1**	**D2**	**D3**	**P value**
LPS (n=8)	23.46±0.3585^a^	20.36±0.3679^b^	19.39±0.4042^b^	<0.0001
LPS+antibiotic (n=8)	24.01±0.4482^a^	20.66±0.4013^b^	19.6±0.3703^b^	<0.0001
Antibiotic (n=8)	24.96±0.9^a^	24.7±0.9165^a^	24.94±0.8664^a^	0.9741

Notes: D1: Body weight of mice before administration on the first day; D2: Body weight of mice before administration on the second day; D3: Body weight of mice before administration on the third day. There was no significant difference between the two groups when marked with the same letter (P > 0.05).

(2) A BL-420 biological signal acquisition system was used to detect the intestinal motility of the mice. We found that (Table 2) there were significant differences in the frequency of intestinal electric waves among the LD3, AD3 and LAD3, while in area, only the AD3 (821.96±339.61) was different from the other two groups. LAD3 had the slowest intestinal motility frequency and the smallest area (347.9±136.24).

**Table 2:** The frequency and area of LD3, LAD3 and AD3 intestinal motility frequency and area were compared.

**Index**	**LD3 (n=8)**	**AD3 (n=8)**	**LAD3 (n=8)**	**P value**
Frequency	0.08 (0.07-0.11)^a^	0.17 (0.12-0.35)^b^	0.05 (0.05-0.06)^c^	0.0002
Area	438.09±130.39^a^	821.96±339.61^b^	347.9±136.24^a^	0.0012

Notes: LD3: The 3rd day after continuous administration of LPS; AD3: the 3rd day after continuous administration of antibiotic; LAD3: The 3rd day after continuous administration of LPS and antibiotic. There was no significant difference between the two groups when marked with the same letter (P > 0.05).

**Figure 2:**
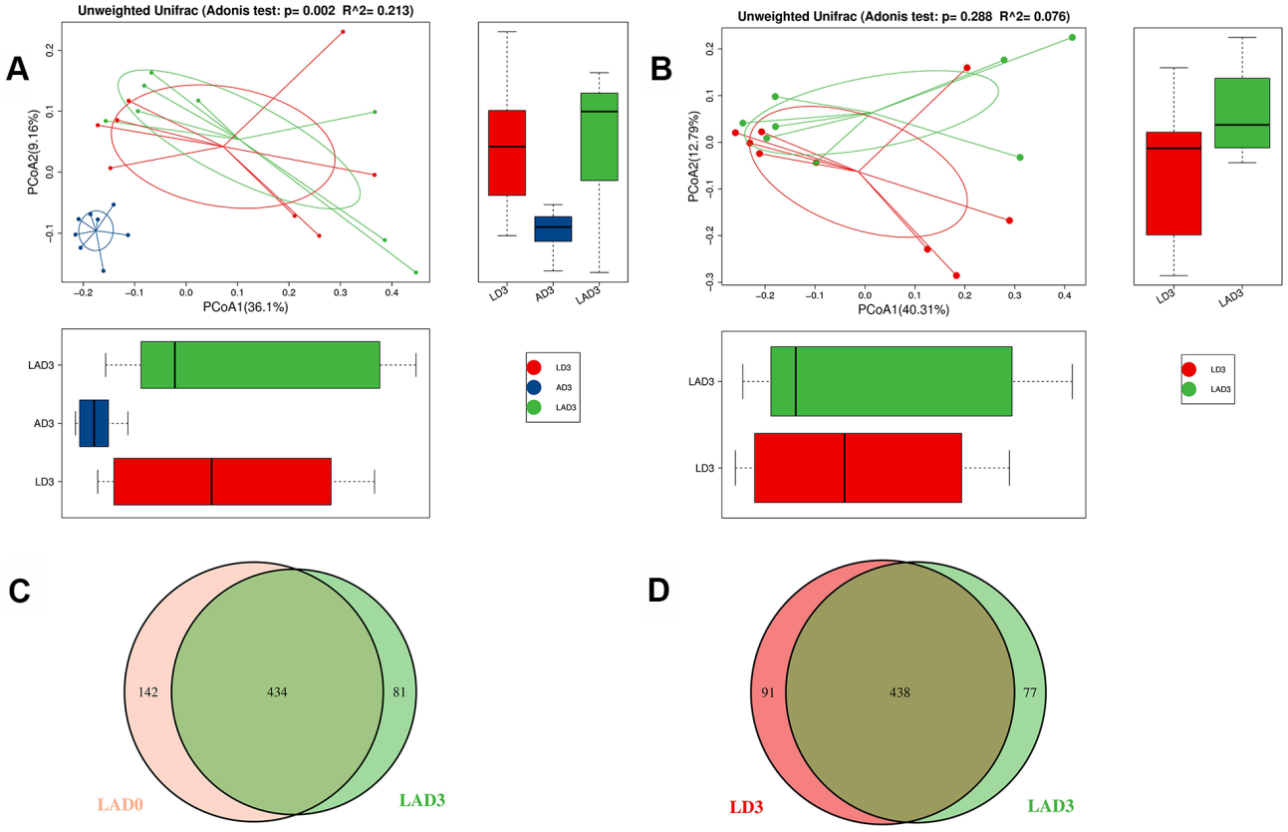
**Adonis analysis was used to compare the difference of beta diversity among LD3, LAD3 and AD3.** OTU Venn diagram was used to calculate the number of common and unique OTUs of LAD0 and LAD3 and LD3 and LAD3. (**A**) Compare LD3, Adonis analysis of species diversity between AD3 and LAD3 groups. (**B**) Adonis analysis of species diversity between LD3 and LAD3 groups. (**C**) OTU Venn analysis of fecal abundance between LAD0 and LAD3 groups. (**D**) OTU Venn analysis of fecal abundance between LD3 and LAD3 groups; P ≤ 0.01 means an extremely significant difference).

**Figure 3:**
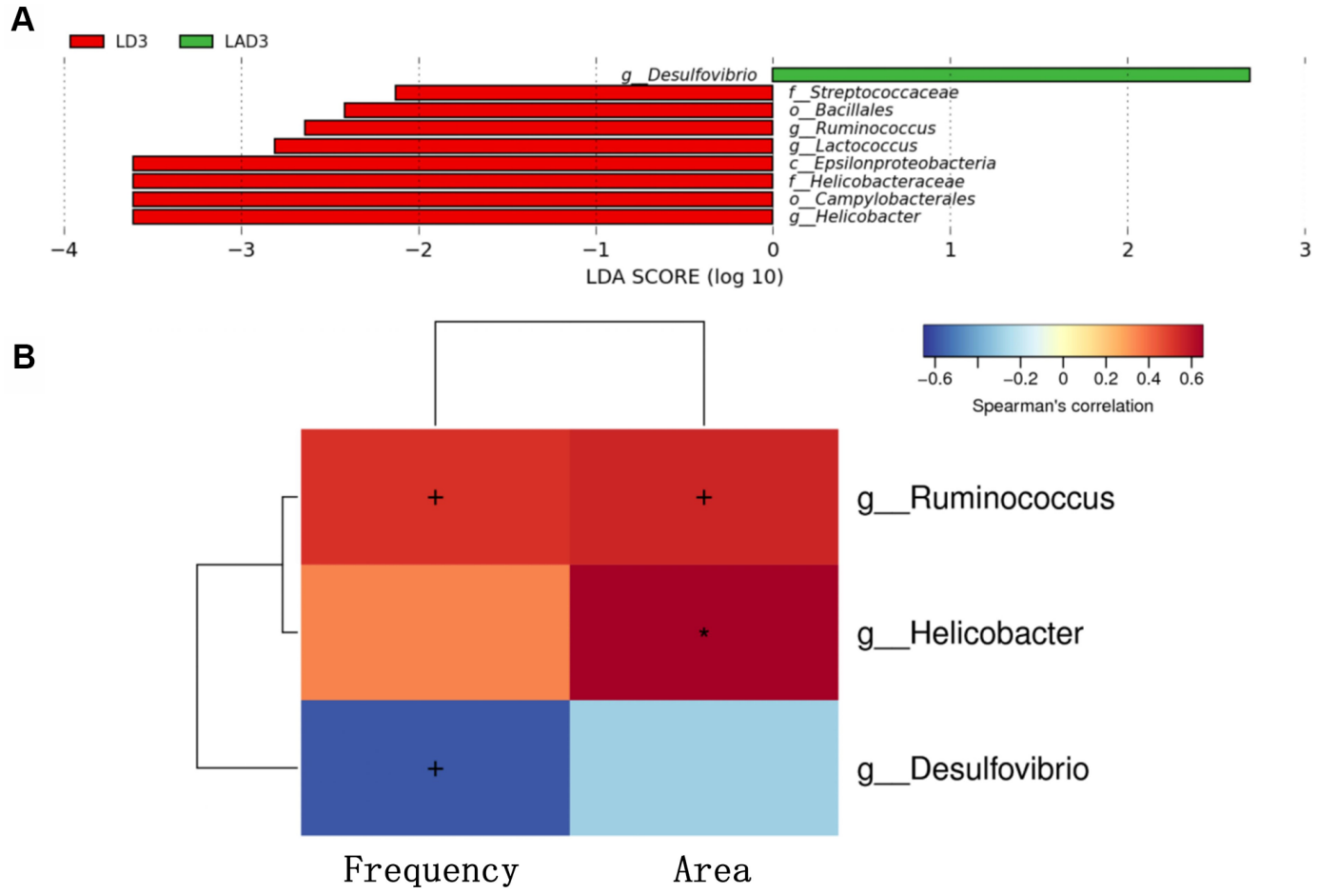
**Linear discriminant analysis effect size (lefse) was used to analyze the species abundance of LD3 and LAD3 groups.** Spearman correlation thermography was used to analyze the correlation between different species and intestinal motility indexes (frequency, area) between LD3 and LAD3 groups. (**A**) Lefse analysis was used to compare the main functional species between LD3 and LAD3 groups. (**B**) Spearman correlation heat map was used to compare the correlation between LD3 and LAD3 groups and the frequency and area of intestinal motility index. *Ruminococcus* and *Helicobacter* were positively correlated with frequency and area, while *Desulfovibrio* was negatively correlated. (p < 0.05, + marks significance, p < 0.01, “*” marks significance).

**Figure 4:**
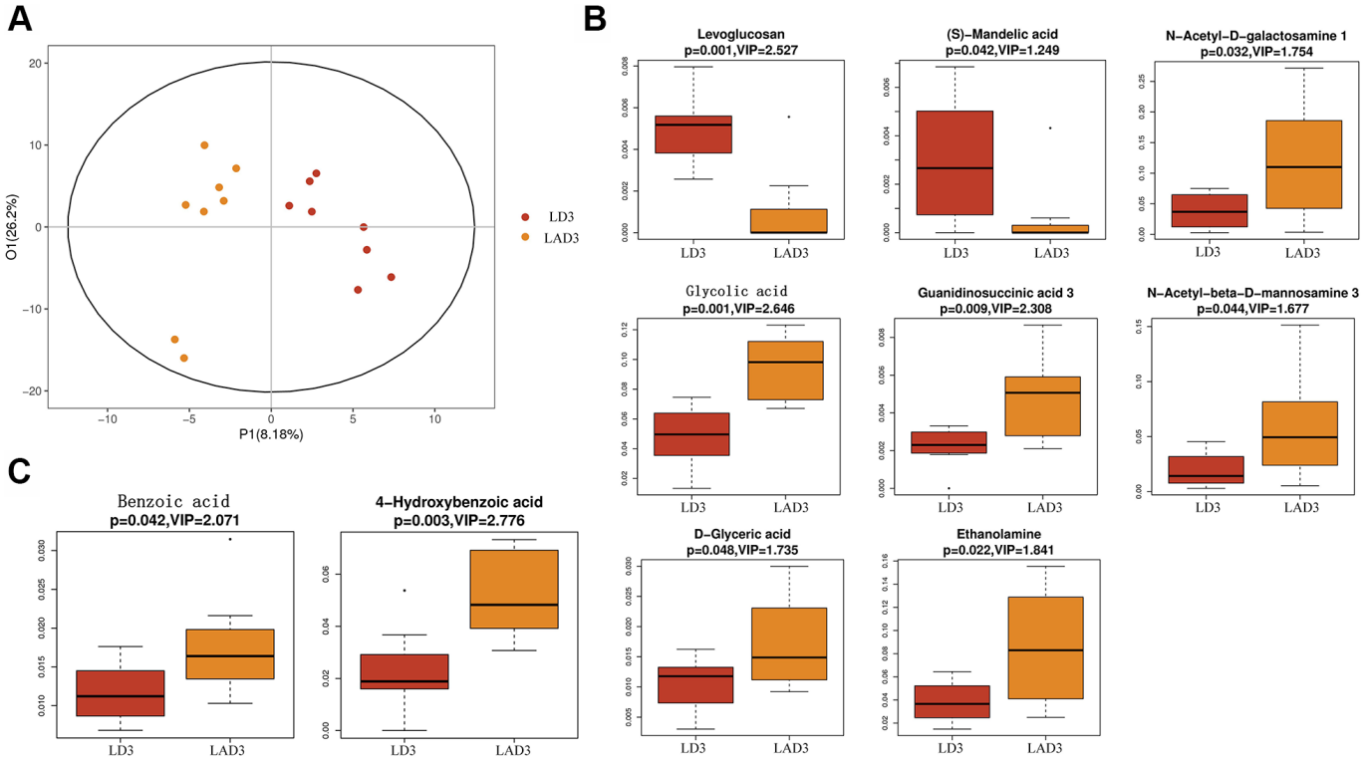
**Orthogonal partial least squares discriminant analysis (OPLS-DA) was used to screen differential metabolites, and boxplot was used to display the results of differential metabolites.** (**A**) The OPLS-DA of LD3 and LAD3 were compared. (**B**) The metabolites of LD3 and LAD3 were Levoglucosan, (s) - Mandelic acid, N-Acetyl-D-galactosamine, Glycolic acid, D-glyceric acid, Ethanolamine, Guaninosuccinic acid 3, N-Acetyl-beta-D-mannosamine 3. (**C**) The metabolites comparing the specific difference between LD3 and LAD3 are Benzoic acid and 4-Hydroxybenzoic acid.

**Figure 5:**
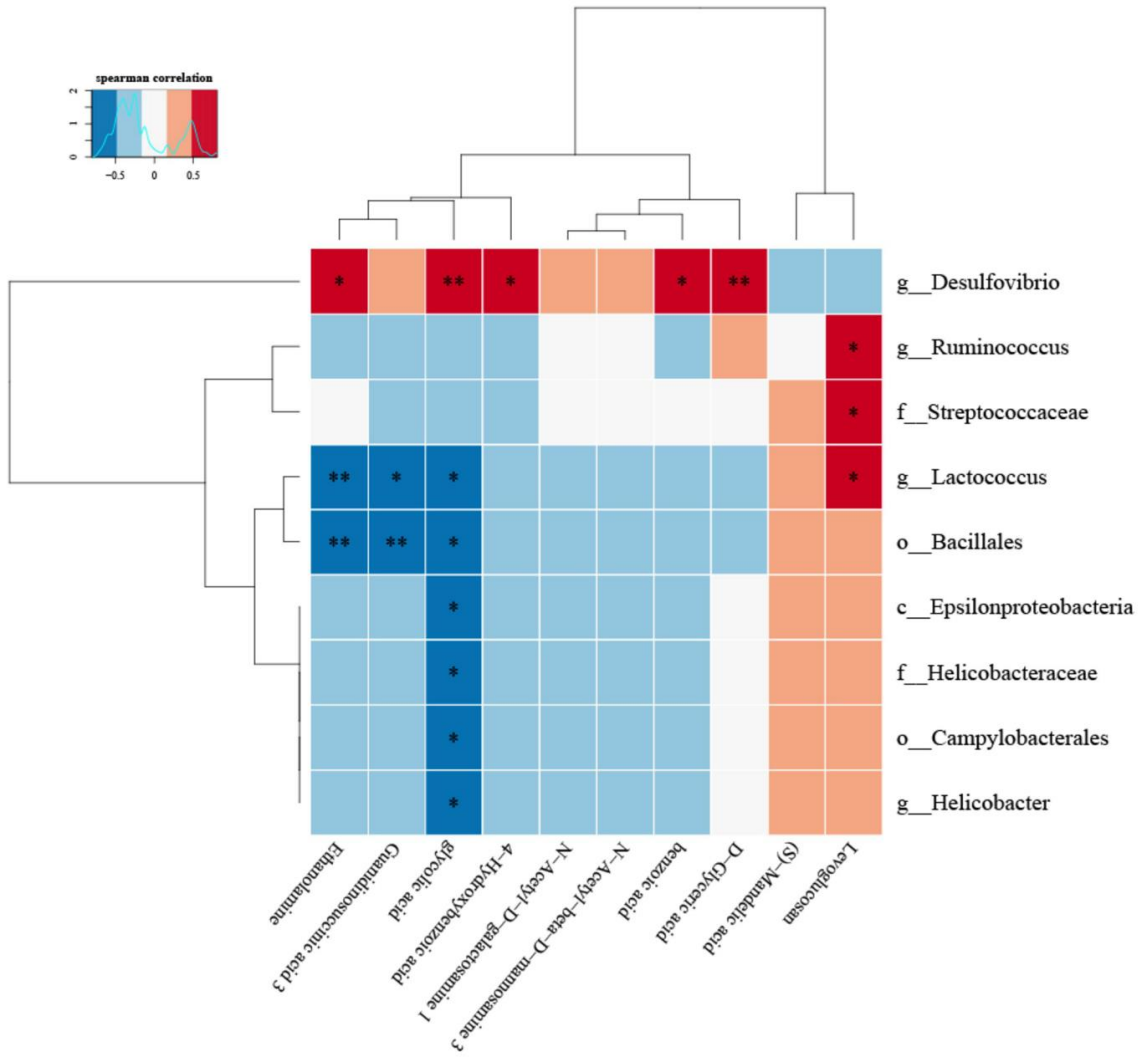
**The correlation between specific metabolites and intestinal microbiota was found by correlation thermogram analysis.** *Desulfovibrio* had a significant positive correlation with Benzoic acid and 4-Hydroxybenzoic acid (P < 0.05). (Below is the difference metabolite, on the right is the difference species, the color depth indicates the correlation size, blue indicates the negative correlation, red indicates the positive correlation, P value is the correlation test result, in the figure, * indicates P < = 0.05, * * = P < = 0.01).

**Figure 6:**
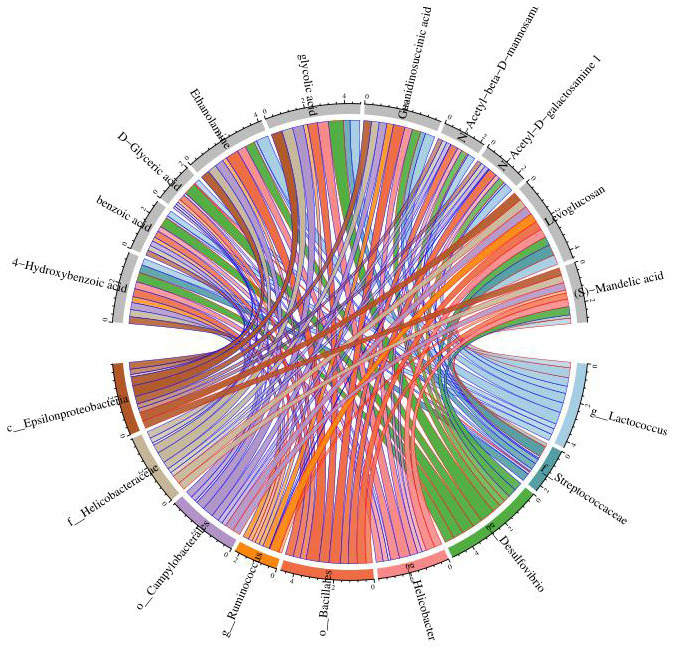
**Chord diagram analysis was used to show the relationship between specific metabolites and intestinal microbiota, and *Desulfovibrio* was positively correlated with benzoic acid and 4-hydroxybenzoic acid.** Nodes represent different species and different metabolites; chord width indicates correlation strength, chord border color indicates correlation, red indicates positive correlation, and blue indicates negative correlation.

(3) The results of Adonis analysis showed that on the third day after the intervention, there were significant differences in bacterial species among the LD3, AD3 and LAD3 (Figure 2A), but there was no significant difference between LD3 and LAD3 (Figure 2B).

(4) Although there was no significant difference between LD3 and LAD3, we found that LD3 and LAD3 displayed different abundances. The bacterial abundance of mice decreased significantly before LAD0 (LPS + antibiotic group before administration) and LAD3 (Figure 2C); However, compared LD3 with LAD3, the abundance of intestinal microbiota decreased after antibiotic treatment (Figure 2D).

(5) LDA effect size (LEfSe): The results showed that the species with a significant difference in LAD3 was *Desulfovibrio*, while the species with significant differences in LD3 were *Streptococcaceae*, *Bacillales*, *Ruminococcus*, *Lactococcus*, *Epsilonproteobacteria*, *Helicobacteraceae*, *Campylobacterales, Helicobacter* (Figure 3A).

(6) The change in intestinal motility frequency and area had a significant correlation with bacteria (Figure 3B). The bacteria with a positive correlation with the frequency and area of intestinal motility included *Ruminococcus* and *Helicobacter*, while the bacteria with a negative correlation were *Desulfovibrio*.

(7) Subsequent untargeted metabolomics analysis showed that the feces of LD3 and LAD3 had different metabolites (Figure 4A). Compared with LD3 group, LAD3 group not only had decreased Levoglucosan and (s) - Mandelic acid, but also increased N-Acetyl-D-galactosamine, Glycolic acid, G-glycoeric acid, Ethanolamine, Guaninosuccinic acid, N-Acetyl-beta-D-mannosamine 3 (Figure 4B), The increase of Benzoic acid and 4-Hydroxybenzoic acid related to specific metabolism was also observed (Figure 4C).

(8) Correlation heat map analysis (Figure 5) and Chord diagram analysis (Figure 6) showed that *Desulfovibrio* was positively correlated with 4-Hydroxybenzoic acid and Benzoic acid.

## DISCUSSION

Sepsis-associated gastrointestinal dysfunction may underlie the pathogenesis of systemic inflammatory response syndrome (SIRS), multiple organ failure (MODS) and septic shock [5, 6, 17]. The gastrointestinal tract is the most easily sacrificed organ in the early stage of sepsis [2, 4]. Dysfunction of intestinal barrier function provides a pathway for intestinal pathogens to enter mesenteric lymph and circulation from the gut [18]. Moreover, intestinal function is easily affected by a variety of factors and is often the organ with the earliest incidence of various diseases [19]. Therefore, the repair and maintenance of the intestinal barrier is a potential target for sepsis treatment.

At present, there are few studies on the influence of antibiotics on the intestinal tract of sepsis patients in the ICU. However, some relevant articles show that the use of broad-spectrum antibiotics can cause changes in the colonic epithelial barrier and the occurrence of inflammatory reactions [9]. The expression of the proinflammatory cytokines IL-1 (interleukin-1), IL-6 (interleukin-6), TNF-α (tumor necrosis factor-α) was significantly increased [9]. At the same time, the thickness of the intestinal epithelial mucus decreased, and the number of apoptotic bodies increased in colon sections. AQP9 (aquaporin Protein-9 Polypeptide) is a channel located mainly in the intestinal tract to manage fecal water, and OCLN (occludin) is the key TJ (tight junction) protein [9, 20] for maintaining intestinal integrity. After 5 days of antibiotic intervention, the expression of TLR4 (toll-like receptor 4) protein and MyD88 (myeloid differentiation primary response gene88) mRNA in the colon was increased, TLR2 (toll-like receptor 2) transcription was decreased, and AQP9 and OCLN levels in the intestinal epithelium of mice were significantly decreased [9]. The effect of antibiotics on AGI in patients with sepsis is unclear, but this treatment will affect the structure of intestinal microbiota and indirectly affect the barrier function of intestinal epithelium [9].

Therefore, to explore the effects of the intestinal microbiota and metabolites on the use of antibiotics during sepsis, we conducted relevant experiments. The results showed that the use of LPS and antibiotics could affect the species and diversity of the intestinal microbiota (Figure 2). When the sepsis mice were treated with antibiotics (LAD3), the inflammatory bacteria *Desulfovibrio* became the dominant bacteria (Figure 3A), which was related to subsequent changes of intestinal motility (Figure 3B). The decrease in intestinal motility may lead to the accumulation of harmful substances. In the feces of sepsis mice (LD3), the dominant bacteria were *Streptococcaceae*, *Bacillales*, *Ruminococcus*, *Lactococcus*, *Epsilonproteobacteria*, *Campylobacterales*, and *Helicobacteraceae.* Among them, *Ruminococcus* and *Helicobacter* had positive effects on the frequency and area of intestinal motility, and the increase of intestinal motility may be the main cause of diarrhea in sepsis mice [21]. We were also found that *Ruminococcus* was positively correlated with Levoglucosan, and *Helicobacter* was negatively correlated with glycolic acid. *Ruminococcus* is a kind of bacteria producing SCFAs [22], which can inhibit intestinal inflammation, provide nutrition for intestinal mucosa and protect intestinal mucosa, so it is considered as a probiotic [23]. The positive metabolite Levoglucosan was associated with the recurrence of Crohn's disease [24]. *Helicobacter* can cause a variety of inflammatory diseases by activating immune response and gastrointestinal disorders [25], and Glycolic acid is an intermediate product of glycolysis. Therefore, we believe that *Ruminococcus* and *Helicobacter* are involved in the process of intestinal inflammation, but it is not clear whether they are positive or negative.

However, *Desulfovibrio*, which is negatively related to intestinal motility, is the only dominant bacteria in the faeces of LAD3, *Desulfovibrio* belong to the SRB (sulfate-reducing bacteria) that can reduce sulfate to the cytotoxic compound hydrogen sulfide. It is also an inflammation-related pathogens [26]. *Desulfovibrio* are not only related to ARGs (antibiotic resistance genes) [27] but also positively correlated with AST (aspartate aminotransferase) and ALT (glutamate pyruvic transa) in serum [28]. The experimental data showed that *Desulfovibrio* increased in the feces of patients with ulcerative colitis [29]. 5-Aminosalicylic acid can inhibit the decrease in sulfate and reduce the level of hydrogen sulfide in feces. This finding suggests that the efficacy of 5-aminosalicylic acid in the treatment of colitis may be due to reduction in the pathogenicity of SRB [30]. *Desulfovibrio* can also degrade and metabolize SCFAs (short-chain fatty acids), which may be the reason for the decrease in SCFAs in mice fed casein or AAs (amino acids) [31–33]. In addition, *Desulfovibrio* can also promote the production of LPS [34, 35], and LPS is the component of the outer wall of gram-negative bacteria that is the main material to induce acute inflammation and sepsis. Overall, these data suggest that *Desulfovibrio* are bacteria that are harmful to the intestinal mucosa and body, and their proliferation may aggravate the occurrence and development of AGI in sepsis.

The results showed that the specific metabolites Benzoic acid and 4-Hydroxybenzoic acid increased in addition to the changes of Levoglucosan, D-glyceric acid and other products involved in the basic metabolism. According to Zellner et al., and Lin et al, the oxidation of Benzaldehyde to Benzoic acid and its derivatives [36] by *Desulfovibrio* was consistent with our results of untargeted metabolomics in feces (Figures 5, 6). Benzaldehyde is the oxidation product of BZA (Benzoylamine). Lin Z showed that BZA could reduce SIRS and stress-associated hyperglycemia induced by LPS in septic mice [37]. LPS could increase the concentrations of plasma NO (nitric oxide) and TNF-α in mice, while intraperitoneal injection of BZA could significantly reduce the concentrations of plasma NO and TNF-α. iNOS (inducible nitric oxide synthase) is essential in the innate immunity and inflammatory response of the host to a variety of pathogens [38]. It catalyzes the production of nitric oxide, a highly active nitrogen free radical, which can reflect the degree of inflammation. COX-2 is expressed in many cells, including macrophages, and produces prostaglandins, leading to inflammatory pain and swelling [39]. In mouse PMs (peritoneal macrophages), BZA caused a decrease in the iNOS and COX-2 expression induced by LPS. Similar results were obtained in mice treated with benzaldehyde [37]. Therefore, in septic mice, *Desulfovibrio* may convert Benzaldehyde, the oxidation product of BZA that inhibits the inflammatory reaction of sepsis, into Benzoic acid. Relevant evidence suggests that Benzylamine may be the intermediate or final metabolite of antibiotics and plays an important role in the antibacterial process of antibiotics.

Therefore, we speculate that the use of antibiotics in septic mice may cause intestinal microecological disorders, resulting in an increase in the inflammation-related pathogenic bacteria *Desulfovibrio*. This increase in bacteria can not only directly act on the intestinal epithelium, resulting in damage to the intestinal epithelium, but also reduce intestinal motility, causing BZA to be converted into benzaldehyde and then oxidized to Benzoic acid [36], which leads to an increase in Benzoic acid and its derivatives in intestinal metabolites.

## CONCLUSIONS

Antibiotics can change the intestinal microbiota and metabolites in sepsis mice, which is related to the frequency and area of the intestinal motility. However, the increase in *Desulfovibrio*, Benzoic acid and 4-Hydroxybenzoic acid in the late stage of antibiotic treatment was associated with the severity of sepsis and AGI. We hope that by inhibiting the production of *Desulfovibrio*, we can reduce the acute gastrointestinal injury, thereby reducing the inflammatory response in sepsis. Benzoic acid and 4-Hydroxybenzoic acid can be used as rapid markers for detection of *Desulfovibrio*. Whether inhibition of *Desulfovibrio* can improve the prognosis of sepsis and whether Benzoic acid and 4-Hydroxybenzoic acid can be used as related markers of *Desulfovibrio* proliferation and the related mechanism of this effect still need to be further studied and further confirmed by experiments.

## MATERIALS AND METHODS

### Animals

Specific pathogen-free (SPF)-grade 4-w-old C57BL/6J (Cancer Research Institute) male mice were provided by Beijing Weitong Lihua Experimental Animal Co., Ltd. Two mice in the same group were placed in the same standard cage for adaptive feeding for 4 weeks. The mice were fed the same feed and purified water (Water equipment, Thermo Scientific, USA) under controlled laboratory conditions (22° C, 12 hour light/dark cycle). Before the experiment, the mice were randomly divided into an experimental group and a control group, and the two groups were kept in different cages.

### Experimental design

Twenty-four 6-8-w-old SPF-grade C57BL/6J male mice were selected, and the mice were randomly divided into three groups, n=8 in each groups: LPS (Lipopolysaccharide, Sigma, USA, from Escherichia coli); antibiotic (Imipenem cilastatin, Hangzhou moshadong Pharmaceutical Co., Ltd); and LPS+antibiotic. The mice were treated by tail vein injection for 3 days, as follows: (1) LPS: 1 mg/kg/day LPS+0.9% NaCl; (2) antibiotic: 25 mg/kg/day imipenem/cilastatin+0.9% NaCl; and (3) LPS+antibiotic: 1 mg/kg/day LPS+25 mg/kg/day imipenem/cilastatin+0.9% NaCl given at the same time. In principle, the total volume of the three treatments was the same, and the total volume was less than 0.2 ml/mouse/time. The insulin needle was 30 gauge×5/16 " (0.3 mm×8 mm). The feces of mice were collected before and 3 days after administration and then stored in sterile cryotubes.

### Sample collection

Fecal collection: (1) To try to ensure the aseptic state in the fecal collection environment, a special plastic box was used for disinfection and drying, and the mice were placed in the plastic box. When the mice entered the unfamiliar environment, they defecated by themselves, and the defecation of mice was be observed at all times. (2) The excreted feces were placed in sterile cryopreservation tubes as soon as possible and marked on ice. (3) The samples were immediately put into a freezer at -80° C.

### Intestinal motility

BL420 bioelectrical signal system (China Taimeng Co., Ltd) was used to detect intestinal motility of mice, and anesthesia machine was used to anesthetize mice. The abdomen of the mice was cut open, and the duodenum 1cm below the pylorus was taken and put into the prepared 37° C desktop liquid (Beijing Solebao Technology Co., Ltd.). The two ends of the intestine were fixed on the BL420 bioelectrical signal machine with No. 4-0 operation line. The intestinal peristalsis in the device was seen, proving that the intestinal tract survived. Then, the link program was used to detect the intestinal motility of mice. The intestinal motility of mice 5-10 minutes after the machine was taken for statistical analysis.

### Microbial community analysis

First, DNA extraction and PCR amplification were carried out. DNA was extracted according to the instructions of the Kit51604 (Shanghai Canspec Scientific Instruments Co., Ltd.). Using primers 341F 5'-CCTACGGGRSGCAGCAG-3' and 806R 5'-GGACTACVVGGGTATCTAATC-3'. The V3-V4 region of bacterial ribosome RNA genes was amplified by PCR. For MiSeq PE300/PE250 or HiSeq PE250 sequencing, the amplicons were extracted from a 2% agarose gel, purified by an AxyPrep DNA Gel Extraction Kit (Axygen Biosciences, Union City, CA, USA) according to the manufacturer's instructions. After the library was prepared, the tags were sequenced on the MiSeq/HiSeq platform (Illumina, Inc., CA, USA). For sequencing data processing, the remaining length and average basic quality of the trimmed barcode and primer tags were further checked. Using uparse (http://drive5.com/UPARSE/), the operational taxonomic units (OTUs) were clustered, and the similarity was 97%. The chimeric sequences were identified and deleted by using usearch (version 7.0). RDP Classifier (http://RDP.cme.msu.edu/) was used according to the RDP database (http://RDP.cme.msu.edu/). The OTU profiling table and α/β diversity analysis were implemented by using the Python script of QIIME.

### Untargeted metabolomics sequencing

Feces were mixed at a ratio of 1:10 (w/V). The mixed solution was swirled for 2 min, then the intestinal and cecal contents were ultrasonically dispersed for 10 min, and fecal samples were centrifuged for 15 min. After centrifuging at 20000 ×g at 4° C for 15 min, the supernatant was transferred to an automatic collection bottle for metabolomics analysis. Five liters of the supernatant was introduced into a Waters Acquity ultra-performance liquid chromatography (UPLC) system (Waters, Milford, MA, USA). The UPLC mobile phase consisted of 0.1% formic acid (solution A) and 0.1% formic acid (solution B). The metabolites were eluted with a linear gradient at a constant flow rate of 0.4 ml/min. The linear gradient was 2-80% solution B, the elution time was 0-15 min, and the elution time of 80-98% solution B was 15-17 min and then returned to 2% solution B at 19.1 min. As mentioned earlier, mass spectrometric data were collected using a Waters QToF Premier mass spectrometer (Waters, Milford, MA, USA) in positive ionization mode. The data were collected with the parameters M/Z 80-1000 over 0-22 min in full scan mode. The original UPLC/MS data were analyzed using micromass markerlynx XS version 4.1 (Waters, Milford, MA, USA) and extended statistical tools. The alignment data from the markerlynx analysis of the QToF MS data were screened using the combined QC samples.

### Enzyme linked immunosorbent assay

ELISA Kit (Jiangsu enzyme immunoassay Industry Co., Ltd.) was used to prepare standard diluent according to concentration gradient. Blank hole, standard hole and sample hole are set respectively. 50ul of standard sample was added to the coated plate, 40ul of sample diluent was added to the well, and then 10ul of sample was added. Try not to touch the hole wall, gently shake and mix. After the plate was sealed with sealing film, it was incubated at 37° C for 30 minutes. Remove the sealing film, discard the liquid and dry it. Fill each well with washing liquid, leave for 30 seconds and discard, repeat for 5 times. 50 UL of enzyme labeled reagent was added to each well, except blank well. Incubate for 30 minutes and wash for 5 times. Add 50 UL of developer a and 50 UL of developer B to each well, mix them gently, and develop in dark at 37° C for 10 minutes. 50 UL of termination solution was added to each well. Zero the blank hole and measure the OD value of each hole at 450nm.

### Statistical analyses

SAS9.4 software was used for statistical analysis, the quantitative data of normal distribution was described with mean ± standard deviation $\left(\bar{x}\pm \;s\right)$, the analysis of variance was used for comparison among the three groups, and the Bonferroni method was used for pairwise comparison of the indicators with statistical significance among the three groups; the quantitative data of skew distribution was described with median and quartile interval [M (P25, P 0.05), Kruskal and Wallis h test were used to compare the three groups; Bonferroni method was used to compare the indicators with statistical significance among the three groups.

### Ethical statement

The authors are accountable for all aspects of the work in ensuring that questions related to the accuracy or integrity of any part of the work are appropriately investigated and resolved. Experiments were performed under a project license (NO.: KY2018-02) granted by regional ethics board of Harbin Medical University Cancer Hospital on December 2018, in compliance with Chinese guidelines for the care and use of animals. The chairperson of the ethics committee is Changhong Zhao.
